# GPI Is a Prognostic Biomarker and Correlates With Immune Infiltrates in Lung Adenocarcinoma

**DOI:** 10.3389/fonc.2021.752642

**Published:** 2021-11-29

**Authors:** Jiahui Han, Xinzhou Deng, Renhuang Sun, Ming Luo, Meng Liang, Bing Gu, Te Zhang, Zhen Peng, Ying Lu, Chao Tian, Yutao Yan, Zhiguo Luo

**Affiliations:** ^1^ Department of Clinical Oncology, Taihe Hospital, Jinzhou Medical University Union Training Base, Shiyan, China; ^2^ Department of Clinical Medicine, The First Clinical College of Hubei University of Medicine, Shiyan, China; ^3^ Department of Clinical Oncology, Taihe Hospital, Hubei University of Medicine, Shiyan, China; ^4^ Hubei Key Laboratory of Stem Cell Research, Taihe Hospital, Hubei University of Medicine, Shiyan, China; ^5^ Department of Oncology, Danjiangkou First Hospital, Danjiangkou, China; ^6^ Biomedical Research Institute, Hubei University of Medicine, Shiyan, China

**Keywords:** glucose-6-phosphate isomerase (GPI), prognosis (carcinoma), immune infiltrate, cell cycle, lung adenocarcinoma

## Abstract

**Background:**

Glucose-6-phosphate isomerase (GPI) plays an important role in glycolysis and gluconeogenesis. However, the role of GPI in lung adenocarcinoma (LUAD) remains unclear.

**Methods:**

All original data were downloaded from The Cancer Genome Atlas (TCGA) and Gene Expression Omnibus (GEO) databases and integrated *via* R 3.2.2. GPI expression was explored with TCGA, GEO, and Oncomine databases. Immunohistochemistry staining was used to analyze GPI expression in clinical specimens. The correlations between GPI and cancer immune characteristics were analyzed *via* the TIMER and TISIDB databases. GPI-specific siRNAs were used to verify the role of GPI expression on cell proliferation and cell cycle distribution.

**Results:**

In general, GPI is predominantly overexpressed and has reference value in the diagnosis and prognostic estimation of LUAD. Upregulated GPI was associated with poorer overall survival, clinical stage, N stage, and primary therapy outcome in LUAD. Mechanistically, we identified a hub gene that included a total of 56 GPI-related genes, which were tightly associated with the cell cycle pathway in LUAD patients. Knockdown of GPI induced cell proliferation inhibition and cell cycle arrest. GPI expression was positively correlated with infiltrating levels of Th2 cells and regulatory T cells (Tregs); in contrast, GPI expression was negatively correlated with infiltrating levels of CD8^+^ T cells, central memory T cells, dendritic cells, macrophages, mast cells, and eosinophils. GPI was negatively correlated with the expression of immunostimulators, such as CD40L, IL6R, and TMEM173, in LUAD.

**Conclusion:**

GPI may play an important role in the cell cycle and can be used as a prognostic biomarker for determining the prognosis and immune infiltration in LUAD.

## Introduction

Lung cancer is one of the malignant cancers with the highest incidence and the worst prognosis worldwide. Lung cancer is divided into small cell lung cancer and non-small-cell lung cancer (NSCLC) (1, 2). Lung adenocarcinoma (LUAD) is the largest subgroup of NSCLC (3). Recently, new therapies have been created for NSCLC, including surgery, chemotherapy, radiotherapy, targeted therapy, and immunotherapy (4). However, the recurrence rate and mortality rate of LUAD are still high, and the prognosis of patients is poor. Therefore, it is imperative to find more effective biomarkers to facilitate novel therapeutic methods.

Immunotherapy has achieved significant results in a variety of tumors and is expected to improve the prognosis of NSCLC (5). However, whether the interaction between NSCLC and the immune system could affect the prognosis of NSCLC patients is largely unclear. Tumor-infiltrating lymphocytes (TILs) have been shown to play an important role in tumor-associated immune responses (6). A number of studies have shown that TILs can effectively improve the prognosis of various tumors (7, 8). Therefore, it is of great clinical significance to investigate the infiltration of immune cells in NSCLC.

Glucose-6-phosphate isomerase (GPI) is a member of the glucose phosphate isomerase protein family. GPI plays an important role in glycolysis and gluconeogenesis, interconverting d-glucose-6-phosphate and d-fructose-6-phosphate (9, 10). GPI can be secreted to the outside of cells, functioning as a cytokine or growth factor (11). GPI is an important autoantigen in human rheumatoid arthritis (RA), and GPI levels, including soluble GPI and GPI immune complexes, are significantly increased in serum and synovial fluid in RA patients (11). In the past decade, aberrant expression of GPI has also been involved in the development and progression of several cancers (12–14). An increasing number of studies have shown that GPI is not secreted by normal cells, but GPI levels are elevated in the serum or urine of patients with malignant tumors. A high expression of GPI in glioblastoma and renal cell carcinoma is associated with poor prognosis. GPI promotes the migration of melanoma cells and the development of invasive breast cancer stem cells and may be involved in cancer metastasis and invasion (9, 15). However, the role of GPI in LUAD has not been systematically explored in multiple aspects.

In recent years, a growing number of platforms and databases have enabled cancer researchers to use multiple sets of data for bioinformatics analysis of cancer. To better understand the role of the GPI gene in NSCLC, we comprehensively evaluated the relationship between GPI expression and the prognosis of LUAD patients through The Cancer Genome Atlas (TCGA), Gene Expression Omnibus (GEO), Oncomine, and Kaplan–Meier plotter databases. In addition, we also studied the correlation between GPI and TILs using the Tumor Immunity Estimation Resource (TIMER) and TISIDB databases. Our results demonstrate that GPI can be used as a biomarker to predict the prognosis and immune infiltration of LUAD patients.

## Materials and Methods

### Patient Datasets

The messenger RNA (mRNA) expression data (including 535 LUAD samples and 59 adjacent nontumor samples) and clinical information were downloaded from TCGA database (https://cancergenome.nih.gov). We also downloaded the following gene expression profiles from the GEO: GSE10072 (including 33 LUAD samples and 33 paired adjacent nontumor samples), GSE32863 (including 58 LUAD samples and 58 paired adjacent nontumor samples), GSE7503 (including 83 LUAD samples and 83 paired adjacent nontumor samples), GSE30219 (including 84 LUAD samples and 14 adjacent nontumor samples), GSE31210 (including 226 LUAD samples), GSE37745 (including 106 LUAD samples), and GSE3141 (including 58 LUAD samples). These were extracted from the GEO database (https://www.ncbi.nlm.nih.gov/geo/) to further validate our results. The Human Protein Atlas (HPA) database (http://www.proteinatlas.org/) was used to verify the expression of GPI in LUAD at the protein level. We obtained the gene amplification and mutation status of GPI through the cBioPortal for Cancer Genomics (http://www.cbioportal.org/).

### Oncomine Database Analysis

The expression level of the GPI gene in various types of cancers was identified in the Oncomine database (https://www.oncomine.org/resource/login.html) (16). Student’s *t*-test was used to compare the transcription levels of the GPI gene in cancer samples with those in normal controls. The cutoff *p*-value and fold change were defined as 0.05 and 1.5, respectively.

### Survival Analysis

The Kaplan–Meier plotter (http://kmplot.com/analysis/) (17) and GEPIA were used to estimate the correlation between GPI expression and the survival rate of different clinical features in LUAD patients, and the hazard ratio (HR) and log-rank *p*-value of the 95% confidence interval were calculated.

### Protein–Protein Interaction Network Analysis

Based on the Kyoto Encyclopedia of Genes and Genomes (KEGG) interaction (18), we constructed a pathway and gene regulatory network. Protein–protein interaction (PPI) data were extracted from the STRING database based on protein interactions and signaling pathways (https://string-db.org), and the network was constructed using Cytoscape 3.7.2 applications.

### Establishment and Evaluation of the Nomograms for LUAD Survival Prediction

In this study, we selected all independent clinicopathological prognostic factors from Cox regression analysis and constructed a contingency table to evaluate the 1-, 3-, and 5-year overall survival (OS) probability of LUAD patients. By comparing the predicted probability of the line chart with the observed actual probability through a calibration curve, the accuracy of the line chart was verified. The overlapping reference lines show that the model is accurate. Receiver operating characteristic (ROC) analysis was used to compare the prediction accuracy of the line chart of the combined model and the line chart of various clinicopathological prognostic factors.

### Functional Enrichment Analysis

To understand the biological processes and pathways that GPI may participate in, we conducted the following analysis. The first 600 genes related to GPI in LUAD and 56 genes related to LUAD survival were obtained from GEPIA (http://gepia.cancer-pku.cn/) (19). These genes were enriched by Gene Ontology (GO) [including biological processes (BP), cellular components (CC), and molecular function (MF)] and KEGG pathway analyses using the Database for Annotation, Visualization and Integrated Discover (DAVID) and visualized by the R package ggplot2. A corrected *p* < 0.05 was determined to be statistically significant.

### LinkedOmics Database Analysis

The LinkedOmics database (http://www.linkedomics.org) (20) contains multiomics data and clinical data for 32 cancer types and a total of 11,158 patients from TCGA project (20). In this study, the LinkedOmics database was used to explore the expression profile of GPI. We explored the GO biological process (GO_BP) and KEGG pathways of GPI and its co-expressed genes by using gene set enrichment analysis (GSEA) in the Link Interpreter module.

### Timer Database Analysis

TIMER (http://www.cistrome.shinyapps.io) (21) is a convenient and accurate online analysis tool that can explore the expression levels of genes in normal and tumor tissues in multiple tumors in TCGA datasets, infer the abundance of tumor-infiltrating immune cells from the gene expression profiles, and evaluate their clinical impact. In this study, the TIMER database was used to analyze the expression levels of genes in normal and tumor tissues in multiple tumors. The deconvolution statistical method in TIMER was used to infer the abundance of tumor-infiltrating immune cells (TIICs) from the gene expression profiles of LUAD samples in TCGA datasets.

### TISIDB Database Analysis

TISIDB is an integrated knowledge base portal that plays a vital role in detecting the interaction between tumors and the immune system (http://cis.hku.hk/TISIDB/) (22). To further clarify the immune correlation of GPI in cancer, we used the “Immunomodulator” module of the TISIDB database to analyze and evaluate the correlation between GPI expression and the levels of immune checkpoint genes. To further study the association between GPI and chemokine/chemokine receptor expression, we evaluated the expression levels of chemokine/chemokine receptors of TIICs through the “chemokine” module.

### Cell Culture and Transfection

The lung adenocarcinoma cell lines H1975 and A549 were purchased from ATCC and cultured in Dulbecco’s modified Eagle’s medium (DMEM) (Gibco, Waltham, MA, USA) containing 10% fetal bovine serum (FBS) (Biological Industries, Israel) in a CO_2_ incubator at 37°C. The cells were grown to ~60% confluency in six-well plates (NEST Biotechnology Co., Ltd., Wuxi, China) 1 day before transfection. We used Lipofectamine 3000 (Invitrogen, San Diego, CA, USA) to transfect the mall interfering RNA (siRNA) sequences according to the manufacturer’s instructions (23). The cells were cultured with serum-free medium supplemented with 10% FBS 6 h post-transfection and then incubated for 24–72 h. The siRNA sequences of GPI in H1975 and A549 cells were used as follows: siRNA negative control (NC) (5′-TTCTCCGAACGTCACGT-3′), siRNA-GPI #1 (5′-CCAAGATGATACCCTGTGA-3′), siRNA-GPI #2 (5′-GTGCTCA AGTGACCTCTCA-3′), and siRNA-GPI #3 (5′-GGCATATCCTGGTGGATTA-3′).

### Cell Proliferative Assay

H1975 and A549 cells transfected with GPI siRNA were placed in a 96-well plate at 3000/well and allowed to adhere overnight. Ten microliters of Cell Counting Kit-8 (CCK-8 solution) was added to each well and incubated for 2 h. A microplate reader (Thermo Fisher Scientific, Shanghai, China) was used to measure the absorbance at 450 nm every day, and the experiment was repeated three times independently (24).

### Western Blot

Lung cancer cells were lysed with protease inhibitor, phosphatase inhibitor, and RIPA cleavage buffer (Thermo Fisher Scientific, Shanghai, China). The protein lysates were separated by sodium dodecyl sulfate polyacrylamide gel electrophoresis (SDS-PAGE) (Servicebio, Wuhan, China) and imprinted on polyvinylidene fluoride (PVDF) membranes (Millipore, Billerica, MA, USA) for analysis (25). Anti-GPI (1:1,000 dilution, #57893; Cell Signaling Technology, Danvers, MA, USA) and anti-GAPDH (1:5,000 dilution, 60004-1-Ig; Proteintech, Wuhan, China) were incubated overnight at 4°C. Horseradish peroxidase (HRP)-conjugated secondary antibodies (1:2,000 dilution; A0181, Beyotime, Shanghai, China) were added for 2 h at room temperature. Western blot analysis was performed with ImageJ software.

### Cell Cycle Analysis

A549 and H1975 cells were cultured in six-well serum-free culture with 1 × 10^5^ cells for 12 h and were transfected with GPI siRNA. The cells were collected after 48 h, washed twice with phosphate-buffered saline (PBS), and then fixed with 70% pre-cooled ethanol at 4°C for 12 h. The cells were stained with RNase A-containing PI buffer (KGA511; Keygen, Jiangsu, China) for 30 min. Flow cytometry (Beckman Coulter Quanta SC System) was used to detect the cell cycle distribution.

### Immunohistochemistry Staining

This study was approved by the Institutional Research Ethics Committee of Taihe Hospital. Forty-six paraffin-embedded lung adenocarcinoma tissues and paracarcinoma tissues were used for immunohistochemistry (IHC) staining. Each tissue was incubated overnight with a primary antibody for GPI (1:1,000 dilution, #94068; Cell Signaling Technology, Danvers, MA, USA) at 4°C. After washing with PBS, each section was incubated with an HRP-labeled goat anti-rabbit secondary antibody (1:2,000 dilution, A0181; Beyotime, Shanghai, China) for 2 h at room temperature. IHC staining was achieved using 3,3′-diaminobenzidine and counterstained with hematoxylin (Beyotime, Shanghai, China). The IHC staining results were analyzed and scored by two pathologists who were blinded to the sources of the clinical samples. ImageJ software was used to analyze the intensity of staining using a semiquantitative integration method.

### Statistical Analysis

Box plot and scatter plot methods were used to detect the expression level of the GPI gene in LUAD patients. The cutoff value of GPI expression was selected as the median method of gene expression. The Wilcoxon signed-rank test and logistic regression were used to analyze the relationship between the clinical features of LUAD and the expression of GPI. Log-rank test was used to test the *p*-value. Single-factor and multivariate Cox analyses were used to screen potential prognostic factors. In all analyses, *, **, and *** indicate *p* < 0.05, *p* < 0.01 and *p* < 0.001, respectively.

## Results

### GPI Is Highly Expressed in LUAD

**Figure 1:**
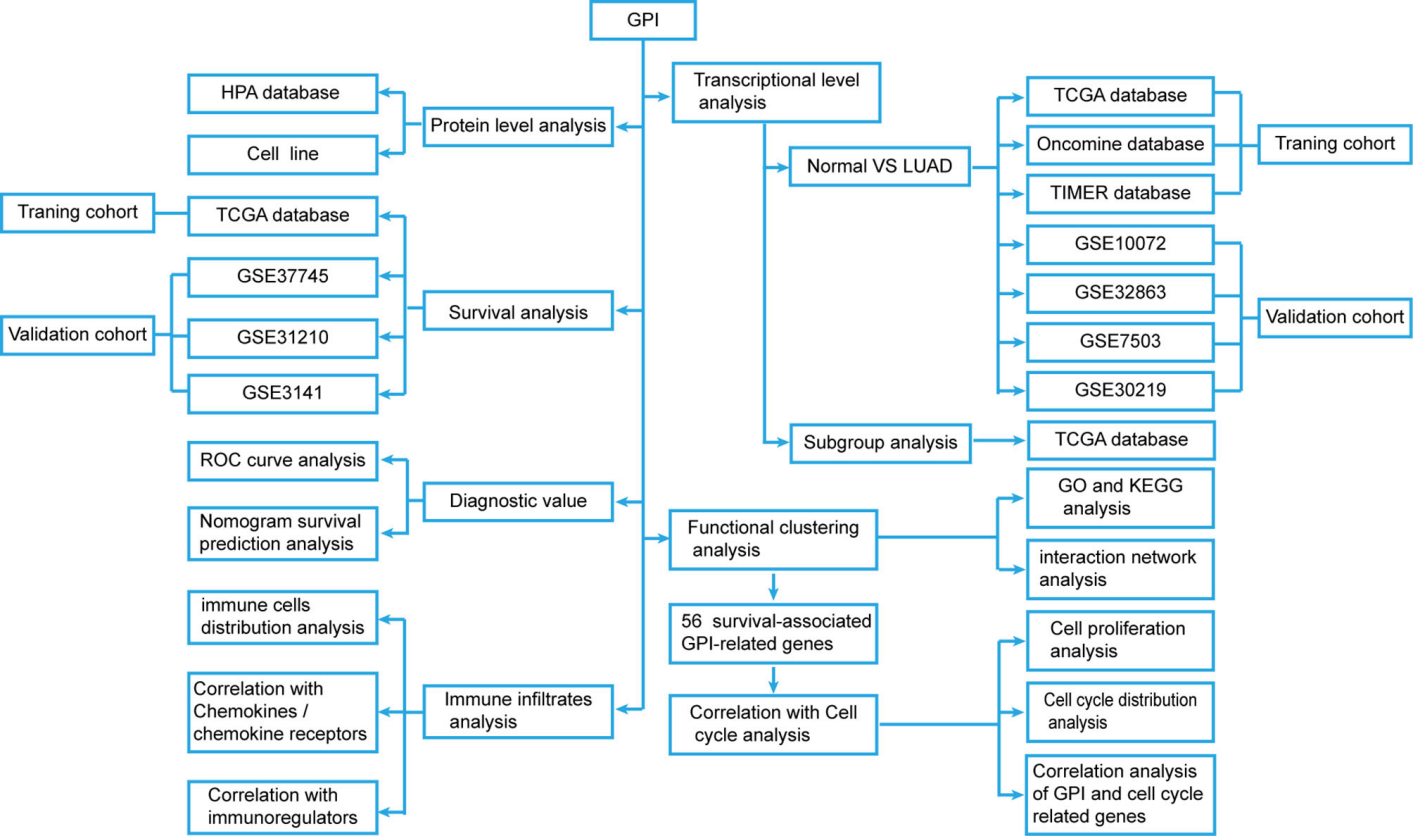
Flow diagram of the data collection and method implementation in this work.

**Figure 2:**
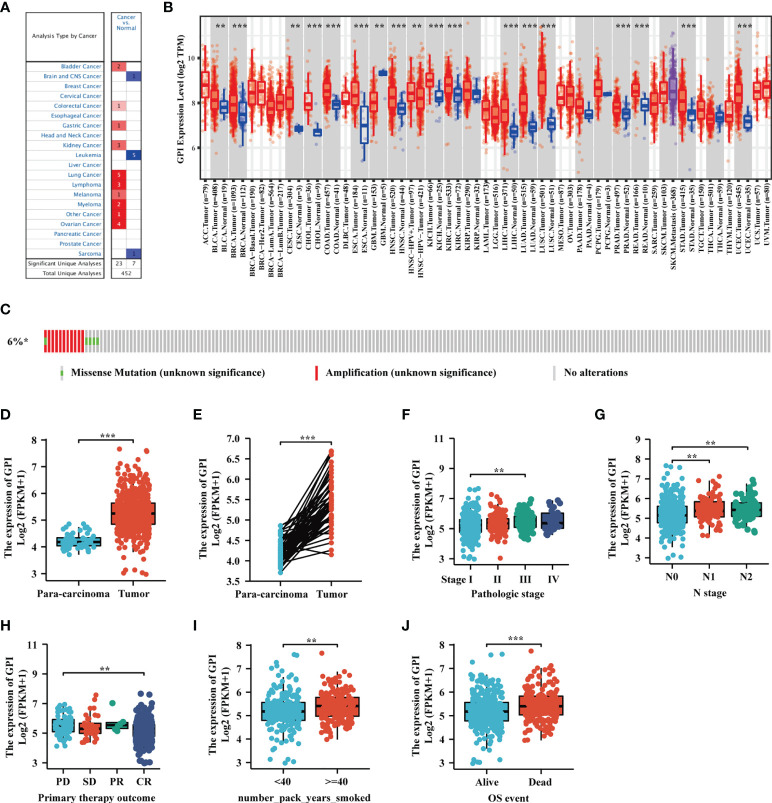
The expression level of glucose-6-phosphate isomerase (GPI) in different human cancers. **(A)** Increased or decreased GPI expression in datasets of different cancers compared with normal tissues in the Oncomine database. **(B)** TIMER was used to detect the expression levels of GPI in different tumors in The Cancer Genome Atlas (TCGA) database. **(C)** The cBioPortal OncoPrint map shows the distribution of GPI genome changes in lung adenocarcinoma (LUAD) patients. **(D–J)** Expression level of GPI in normal tissues and paired adjacent tissues [unmatched tissues **(D)** and matched tissues **(E)**] and the tumor tissues from patients with different clinical characteristics in TCGA [clinical stage **(F)**, N stage **(G)**, primary therapy outcome **(H)**, smoking pack-years **(I)**, and OS event **(J)**]. **p < 0.01, ***p < 0.001.

We developed a flow diagram to show our process (**Figure 1**). To explore the expression level of GPI in normal and tumor tissues, we used the Oncomine database to analyze the expression levels of GPI mRNA in different tumors and normal tissues. The results showed that, compared with that in normal tissues, GPI expression was significantly higher in tumor tissues such as bladder cancer, colorectal cancer, gastric cancer, kidney cancer, lung cancer, ovarian cancer, and lymphoma. In addition, a lower expression was observed in brain tumors, central nervous system (CNS) tumors, leukemia, and sarcoma (**Figure 2A**). Similar results were also obtained from the TIMER database of TCGA clinical patients (**Figure 2B**). In addition, to understand the mutation level of GPI in LUAD, we analyzed its genome and copy number. The OncoPrint map of the GPI gene of LUAD patients in TCGA dataset was analyzed using a cBioPortal map (**Figure 2C**), and the results showed that GPI had less than 6% gene amplification and missense mutations.

We used TCGA database to evaluate the mRNA expression levels of GPI in LUAD patients and compared these with the expression levels in adjacent tissues. The results showed that the expression level of GPI in LUAD was significantly higher than that in paracarcinoma tissues (*p* < 0.01) (**Figure 2D**). The results were verified in LUAD and paired paracarcinoma tissues (**Figure 2E**).

To analyze the correlation between GPI expression and clinical characteristics in LUAD patients, we analyzed the mRNA expression levels of GPI in different clinical categories in TCGA database. The association identified between GPI expression and clinical features in patients with LUAD is summarized in **Table 1**. The results showed that the high expression of GPI was significantly related to the clinical stage (*p* < 0.05), N stage (*p* < 0.05), initial treatment result (*p* < 0.05), and OS event (*p* < 0.01) of these patients (**Figures 2F–J** and **Table 1**). To further elucidate the abnormal upregulated mechanisms of GPI in LUAD tissues, we also explored the correlation of the expression levels of GPI and their methylation status using public databases (DiseaseMeth, version 2.0). The data showed that the difference in the methylation levels between tumor tissues and paracarcinoma tissues was not significant (**Supplementary Table S1**). These data suggest that GPI is significantly upregulated in LUAD.

**Table 1:** Correlation between glucose-6-phosphate isomerase (GPI) expression and the clinicopathological features of the lung adenocarcinoma (LUAD) cases from The Cancer Genome Atlas (TCGA).

Characteristic	Low expression of GPI	High expression of GPI	*p*-value
*n*	267	268	
T stage, *n* (%)			0.100
T1	101 (19)	74 (13.9)	
T2	134 (25.2)	155 (29.1)	
T3	22 (4.1)	27 (5.1)	
T4	9 (1.7)	10 (1.9)	
N stage, *n* (%)			<0.001
N0	193 (37.2)	155 (29.9)	
N1	36 (6.9)	59 (11.4)	
N2	27 (5.2)	47 (9.1)	
N3	0 (0)	2 (0.4)	
M stage, *n* (%)			0.408
M0	183 (47.4)	178 (46.1)	
M1	10 (2.6)	15 (3.9)	
Pathologic stage, *n* (%)			0.003
Stage I	167 (31.7)	127 (24.1)	
Stage II	53 (10.1)	70 (13.3)	
Stage III	31 (5.9)	53 (10.1)	
Stage IV	11 (2.1)	15 (2.8)	
Primary therapy outcome, *n* (%)			0.007
PD	25 (5.6)	46 (10.3)	
SD	17 (3.8)	20 (4.5)	
PR	1 (0.2)	5 (1.1)	
CR	181 (40.6)	151 (33.9)	
Gender, *n* (%)			0.129
Female	152 (28.4)	134 (25)	
Male	115 (21.5)	134 (25)	
Age (years), *n* (%)			0.726
≤65	126 (24.4)	129 (25)	
>65	134 (26)	127 (24.6)	
Residual tumor, *n* (%)			0.386
R0	183 (49.2)	172 (46.2)	
R1	5 (1.3)	8 (2.2)	
R2	1 (0.3)	3 (0.8)	
number_pack_years_smoked, *n* (%)			0.002
<40	104 (28.2)	84 (22.8)	
≥40	70 (19)	111 (30.1)	
Smoker, *n* (%)			0.130
No	44 (8.4)	31 (6)	
Yes	216 (41.5)	230 (44.1)	
Age, median (IQR)	66.5 (59–72.25)	65 (59–72)	0.677

PD, progressive disease; SD, stable disease; PR, partial response; CR, complete response; IQR, interquartile range.

### Validation Using Independent External Databases and Clinical Specimens

**Figure 3:**
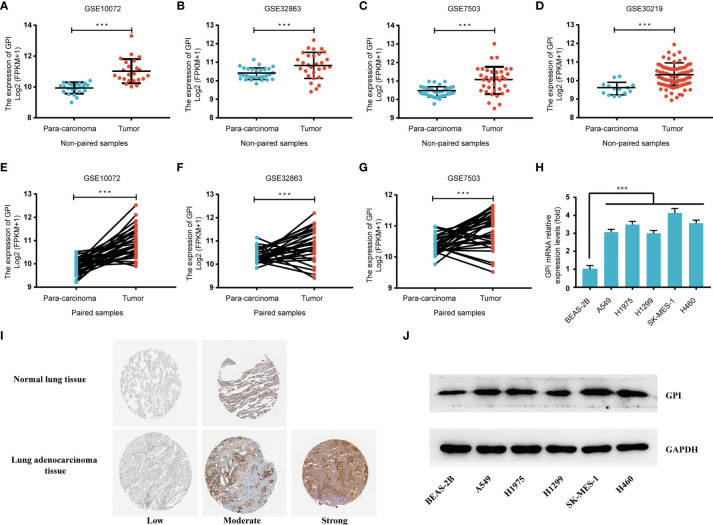
Validation of the high expression of glucose-6-phosphate isomerase (GPI) in lung adenocarcinoma (LUAD) using independent external databases and cell lines. **(A–D)** Expression of GPI in tumor and unpaired paracarcinoma tissues of the GSE10072, GSE32863, GSE7503, and GSE30219 datasets in the Gene Expression Omnibus (GEO) database. **(E–G)** Expression level of GPI in tumor and paired adjacent tissues in the GSE10072, GSE32863, and GSE7503 datasets of the GEO database. **(H)** Transcriptional level of GPI in different non-small-cell lung cancer (NSCLC) cell lines. (**I**) Validation of the expression level of GPI in LUAD using the Human Protein Atlas database (immunohistochemistry). **(J)** Western blot detecting the protein expression level of GPI in different NSCLC cell lines. ***p < 0.001.

To further verify the expression level of GPI in LUAD, we selected another four independent external GEO datasets (validation cohort) to analyze the GPI transcription levels of cancer tissues and adjacent tissues in LUAD, including GSE10072, GSE32863, GSE7503, and GSE30219. The results showed that the transcription level of GPI in LUAD was significantly higher than that in noncancerous adjacent tissues from four LUAD datasets (*p* < 0.001) (**Figures 3A–D**). The results of this comparison were also verified in LUAD tissues and accurately matched noncancerous tissues (GSE30219 does not contain accurately matched noncancerous tissues) and lung cancer cell lines (**Figures 3E–H**).

**Figure 4:**
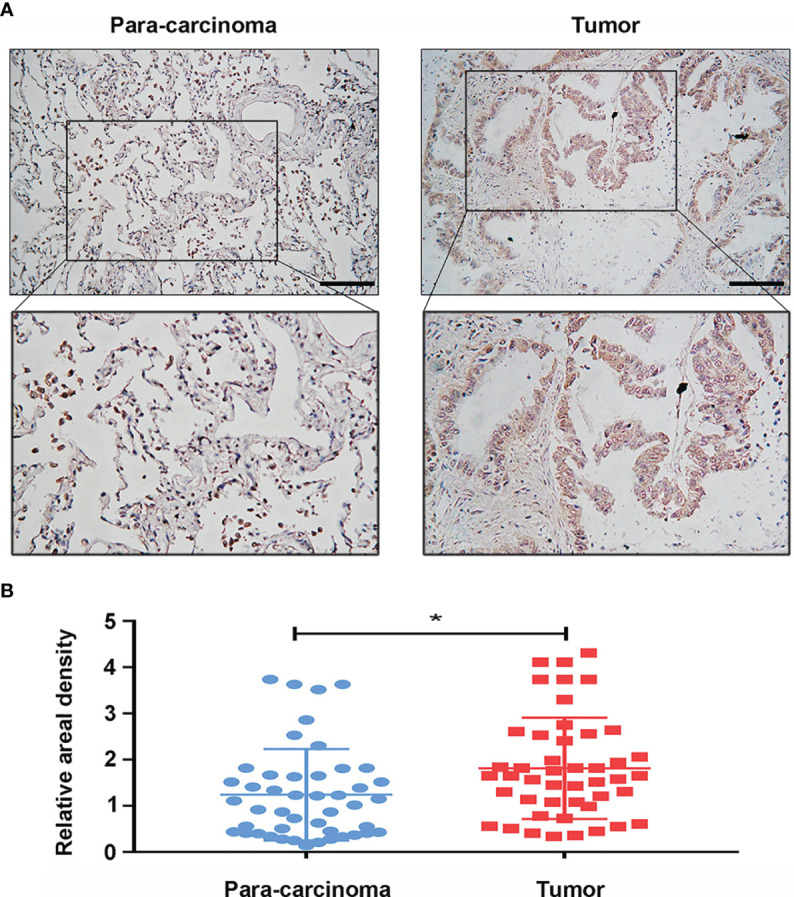
Protein expression of glucose-6-phosphate isomerase (GPI) in clinical specimens of lung adenocarcinoma (LUAD). **(A)** Immunohistochemical staining of GPI was performed in tumor tissues (*n* = 46) and paracarcinoma tissues (*n* = 46). Representative images are shown. *Score bars*, 50 μm. **(B)** Staining was quantified, as shown. The *dot plot* depicts the means and standard deviation of 46 images of tumor tissues and adjacent normal tissues. *p < 0.05.

At the same time, we analyzed the protein expression level of GPI in LUAD based on the HPA database. The data showed that the expression level of GPI in tumors was higher than that in adjacent tissues (**Figure 3I**). In addition, the expression level of GPI in NSCLC cell lines was detected by Western blotting. The results showed that the expression level of GPI in A549, H1975, H1299, SK-MES-1, and H460 cells was significantly higher than that in the normal lung epithelial cell line BEAS-2B (**Figure 3J**). Finally, clinical specimens of LUAD were collected to investigate GPI expression, and the results indicated that the expression level of GPI in tumors was higher than that in paracarcinoma (**Figures 4A, B** **)**. These data verify that GPI is highly expressed in LUAD.

### High Expression of GPI Is an Independent Prognostic Factor for the Overall Survival of LUAD

**Figure 5:**
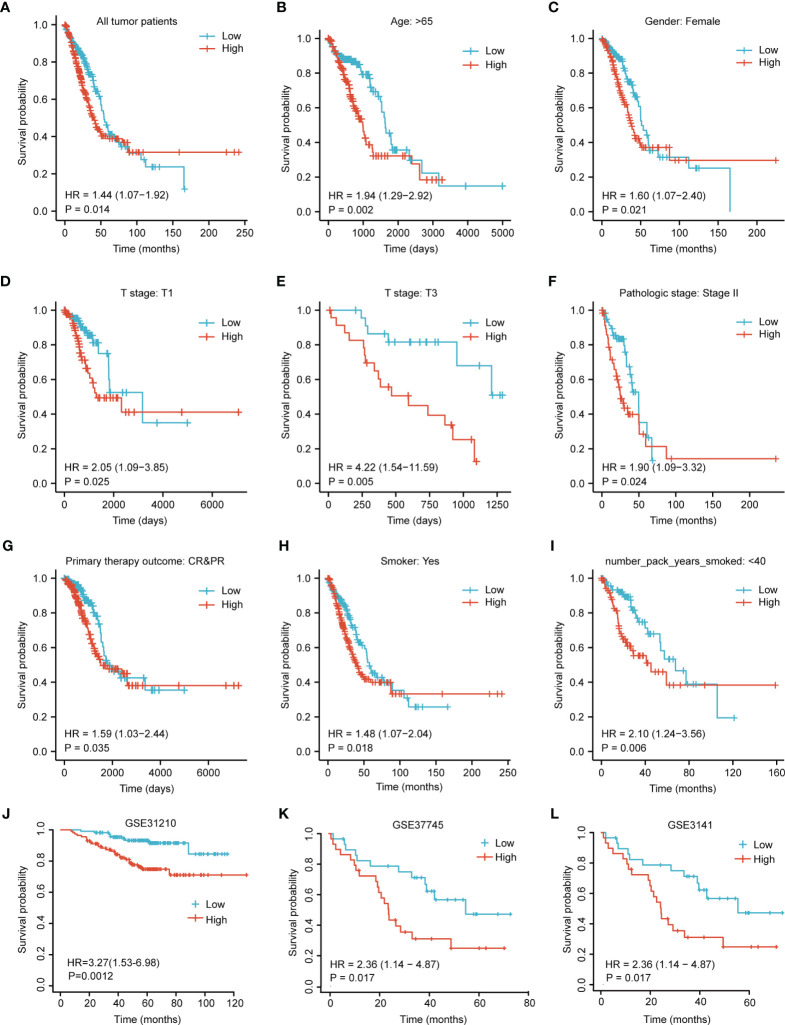
Kaplan–Meier survival curve analysis of the prognostic significance of a high and a low expression of glucose-6-phosphate isomerase (GPI) in lung adenocarcinoma (LUAD) using The Cancer Genome Atlas (TCGA) and Gene Expression Omnibus (GEO) databases. **(A)** Kaplan–Meier estimates of the overall survival probability of TCGA patients in all LUAD patients. **(B–L)** Subgroup analysis for age greater than 65 years **(B)**, female patients **(C)**, T1 **(D)**, T3 **(E)**, pathological staging stage II **(F)**, complete/partial response (CR/PR) **(G)**, smokers **(H)**, and smokers over 40 years old **(I)** and tumor patients in the GEO datasets: GSE31210 **(J)**, GSE37745 **(K)**, and GSE3141 **(L)**.

To identify whether GPI expression affects patient survival, we classified LUAD patients in TCGA database into a high GPI expression group (the top 50% of samples with the highest expression) and a low GPI expression group (the remaining 50% of the samples) in order to perform survival analysis according to the mean expression value of GPI. The Kaplan–Meier survival analysis showed that the high expression of GPI was related to the poor prognosis of LUAD patients (HR = 1.44, *p* = 0.014) (**Figure 5A**). Subgroup analysis showed that a high GPI expression was significantly correlated with poor prognosis in LUAD in the following cases: patients over 65 years old (HR = 1.94, *p* = 0.002), female patients (HR = 1.60, *p* = 0.021), T1 (HR = 2.05, *p* = 0.25), T3 (HR = 4.22, *p* = 0.005), pathological stage II (HR = 1.90, *p* = 0.024), CR/PR (complete/partial response; HR = 1.59, *p* = 0.035), smokers (HR = 1.48, *p* = 0.018), and smokers over 40 years old (HR = 2.10, *p* = 0.006). These data are shown in **Figures 5B–I**.

In addition, we used three other independent external GEO datasets—GSE31210, GSE37745, and GSE3141—as the validation cohort to verify the reproducibility and transferability of GPI expression data in the prognosis of LUAD patients. The Kaplan–Meier survival analysis showed that patients with increased GPI expression in three independent external datasets had shorter OS (**Figures 5J–L**). Univariate Cox analysis demonstrated that a high GPI expression was significantly correlated with poor OS (HR = 1.435, 95% CI = 1.075–1.916, *p* = 0.014) (**Table 2**). These data suggest that a high expression of GPI is an independent prognostic factor for the OS of LUAD patients.

**Table 2:** Univariate and multivariate Cox regression analyses of the clinical characteristics associated with overall survival in lung adenocarcinoma (LUAD) in The Cancer Genome Atlas (TCGA).

Characteristics	Total (*N*)	Univariate analysis		Multivariate analysis	
		Hazard ratio (95% CI)	*p*-value	Hazard ratio (95% CI)	*p*-value
Gender (male *vs.* female)	526	1.070 (0.803–1.426)	0.642		
Age (>65 *vs.* ≤65)	516	1.223 (0.916–1.635)	0.172		
T stage (T3/T4 *vs.* T1/T2)	523	2.317 (1.591–3.375)	**<0.001**	1.847 (0.894–3.818)	0.098
N stage (N1/N2/N3 *vs.* N0)	510	2.601 (1.944–3.480)	**<0.001**	1.551 (0.942–2.556)	0.085
M stage (M1 *vs.* M0)	377	2.136 (1.248–3.653)	**0.006**	1.480 (0.553–3.958)	0.435
Pathologic stage (stage III/stage IV *vs.* stage I/stage II)	518	2.664 (1.960–3.621)	**<0.001**	1.518 (0.770–2.993)	0.228
Primary therapy outcome (PD/SD *vs.* PR/CR)	439	2.653 (1.888–3.726)	**<0.001**	2.825 (1.718–4.646)	**<0.001**
Residual tumor (R1/R2 *vs.* R0)	363	3.879 (2.169–6.936)	**<0.001**	3.272 (1.250–8.567)	**0.016**
Smoker (yes *vs.* no)	512	0.894 (0.592–1.348)	0.591		
number_pack_years_smoked (≥40 *vs.* <40)	363	1.073 (0.753–1.528)	0.697		
GPI (high *vs.* low)	526	1.435 (1.075–1.916)	**0.014**	1.104 (0.705–1.730)	0.665

PD, progressive disease; SD, stable disease; PR, partial response; CR, complete response; GPI, glucose-6-phosphate isomerase; Bold values: P <0.05.

### Diagnostic Value of GPI Expression in LUAD

**Figure 6:**
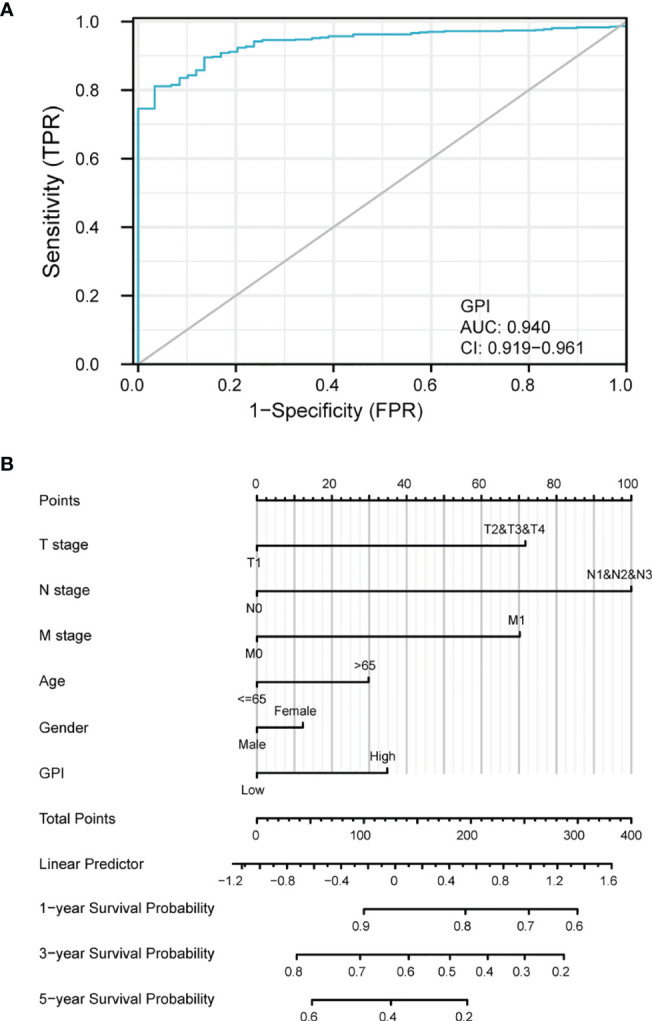
Diagnostic value of glucose-6-phosphate isomerase (GPI) expression in lung adenocarcinoma (LUAD). **(A)** Receiver operating characteristic (ROC) curve analysis for GPI expression in LUAD and adjacent tissue. **(B)** Nomogram survival prediction chart for predicting the 1-, 3-, and 5-year overall survival rates.

To analyze the diagnostic value of GPI expression in LUAD, we performed ROC curve and nomogram analysis on the GPI gene expression data of TCGA database to evaluate the diagnostic value of the gene. The area under the ROC curve (AUC) was 0.940, suggesting a higher diagnostic value, as shown in **Figure 6A**. We combined the expression level of GPI with the clinical variables to construct a nomogram in order to predict the survival probability of patients at 1, 3, and 5 years. The nomogram indicated that the prognostic prediction of the expression level of GPI was better than that of the traditional clinical features of age and sex (**Figure 6B**).

### GPI Is Closely Related to LUAD Cell Cycle Regulation

**Figure 7:**
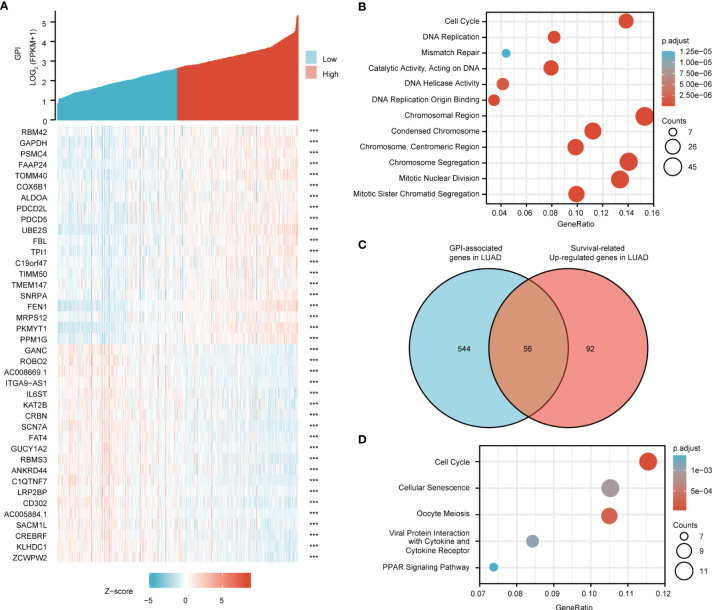
Glucose-6-phosphate isomerase (GPI) functional clustering and interaction network analysis of GPI-related genes. **(A)** Heatmap showing the top 50 genes in lung adenocarcinoma (LUAD) that were positively and negatively related to GPI. *Red* represents positively related genes and *blue* represents negatively related genes. **(B)** Gene Ontology (GO) term and Kyoto Encyclopedia of Genes and Genomes (KEGG) pathway analyses of GPI-related genes in LUAD. **(C)** Venn diagram of the GPI-related genes and the survival-related and upregulated genes in LUAD. **(D)** GO term and KEGG pathway analyses of the GPI-related genes and LUAD survival-related genes in LUAD.

To understand the biological function of GPI in LUAD, we used the LinkFinder module of the LnkedOmics website to detect the co-expression pattern of GPI in LUAD of TCGA database. The red dot indicates the top 25 genes that were positively correlated with GPI, and the green dot represents the bottom 25 genes that were negatively correlated with GPI (**Figure 7A**).

We used DAVID Functional Annotation Bioinformatics Microarray Analysis to identify the enriched GO functional enrichment and KEGG pathways among the GPI-related genes (top 600) and found that cell cycle signaling pathways, RNA replication, and mismatch repair were enriched among these genes (**Figure 7B**).

To identify genes with the same regulatory direction in high-GPI and non-survival patients, we crossed the 600 genes with the highest GPI correlation with the 148 survival-related upregulated genes in LUAD and detected 56 genes at the intersection that were related to GPI and LUAD survival (**Figure 7C**). These 56 protein-coding genes may be potential genetic biomarkers for LUAD patients. GO functional enrichment and KEGG pathway analysis were performed for these 56 genes, and the results showed that differentially expressed genes (DEGs) were significantly enriched in the cell cycle from the GO terms and KEGG pathways (**Figure 7D**).

**Figure 8:**
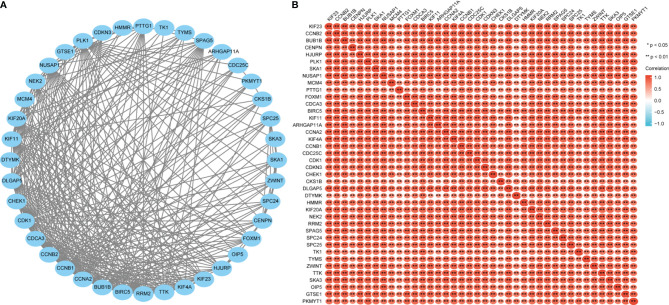
Glucose-6-phosphate isomerase (GPI)-related gene interaction network **(A)** and gene co-expression matrix **(B)**.

After discovering significantly different pathways, PPIs and correlated analysis were used to identify the interactions between these 56 proteins. We found that there was a stronger enrichment network among these proteins than in random proteins, and these genes were particularly related to cell cycle pathways (**Figure 8A**). Gene co-expression correlation analysis showed that most of the proteins in the network had a strong positive correlation with each other (**Figure 8B**). Therefore, these GPI-associated established genes, especially cell cycle-related genes, have a strong intertwined interaction and can be used as multigene biomarkers to predict the survival of LUAD patients.

### GPI Affects LUAD Cell Proliferation by Regulating the Cell Cycle

**Figure 9:**
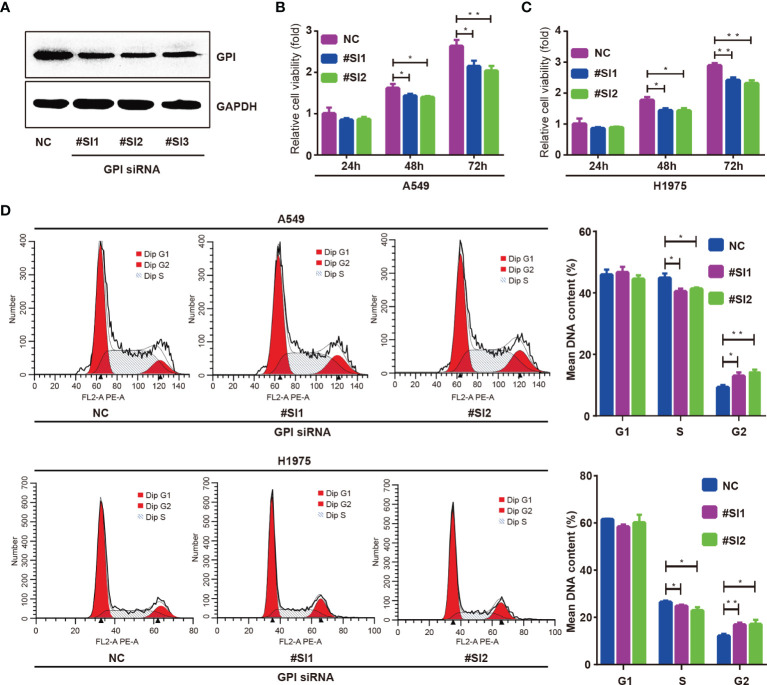
Glucose-6-phosphate isomerase (GPI) regulates the cycle progression of lung adenocarcinoma (LUAD) cells. **(A)** Western blot detection of the interfering ability of GPI siRNA in A549 cells. A549 and H1975 cells were transfected with GPI siRNA #SI1 and #SI2. **(B–D)** CCK-8 was used to detect the proliferation ability of A549 cells **(B)** and H1975 cells **(C)**, and flow cytometry was used to detect the cell cycle distribution of A549 and H1975 cells **(D)**. *p < 0.05, **p < 0.01.

To explore the regulatory effect of GPI on the cell cycle, GPI-targeted siRNA sequences were used to interfere with GPI expression, and the knockout efficiency was detected by Western blotting. The results showed that the expression of GPI was significantly decreased after transfection of GPI siRNA (**Figure 9A**). CCK-8 assays were applied to investigate the effect of GPI on cell proliferation. The results showed that silencing GPI significantly inhibited the proliferation of A549 and H1975 cells (**Figures 9B, C** **)**. The effect of silencing GPI on the cell cycle of LUAD cells was further determined by flow cytometry. The results showed that A549 and H1975 cells revealed G2/M cell cycle arrest after GPI knockdown (**Figure 9D**). These data indicated that GPI affects the proliferation of LUAD cells by regulating cell cycle progression.

### GPI Is Closely Related to Cell Cycle Regulatory Genes in LUAD

**Figure 10:**
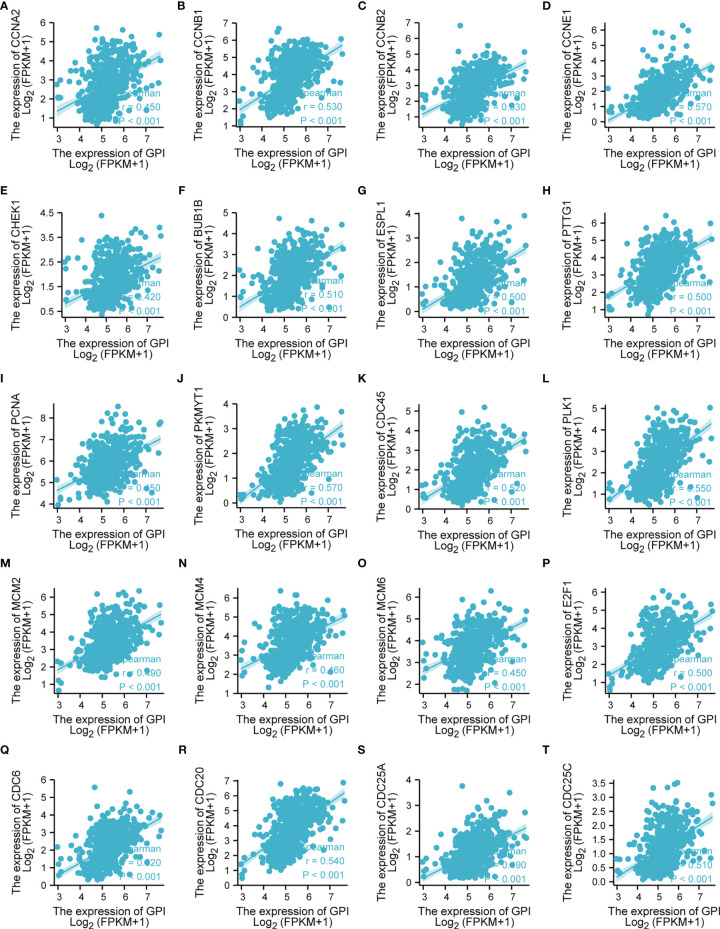
Correlation analysis between glucose-6-phosphate isomerase (GPI) and the cell cycle regulatory genes in lung adenocarcinoma (LUAD) in The Cancer Genome Atlas (TCGA). **(A)** CCNA2, **(B)** CCNB1, **(C)** CCNB2, **(D)** CCNE1, **(E)** CHEK1, **(F)** BUB1B, **(G)** ESPL1, **(H)** PTTG1, **(I)** PCNA, **(J)** PKMYT1, **(K)** CDC45, **(L)** PLK1, **(M)** MCM2, **(N)** MCM4, **(O)** MCM6, **(P)** E2F1, **(Q)** CDC6, **(R)** CDC20, **(S)** CDC25A, and **(T)** CDC25C.

The above bioinformatics analysis indicated that GPI is significantly enriched in the cell cycle pathway. Therefore, we further analyzed the correlation between GPI and the cell cycle regulation genes in TCGA-LUAD, and we found that the expressions of the cell cycle regulatory genes *CCNA2*, *CCNB1*, *CCNB2*, *CCNE1*, *CHEK1*, *BUB1B*, *ESPL1*, *PTTG1*, *PCNA*, *PKMYT1*, *CDC45*, *PLK1*, *MCM2*, *MCM4*, *MCM6*, *E2F1*, *CDC6*, *CDC20*, *CDC25A*, and *CDC25C* were positively correlated with GPI (*r* > 0.3, *p* < 0.001) (**Figures 10A–T**). These data verify that GPI is closely related to LUAD cell cycle regulation genes.

### Correlation of GPI Expression With Immune Characteristics

**Figure 11:**
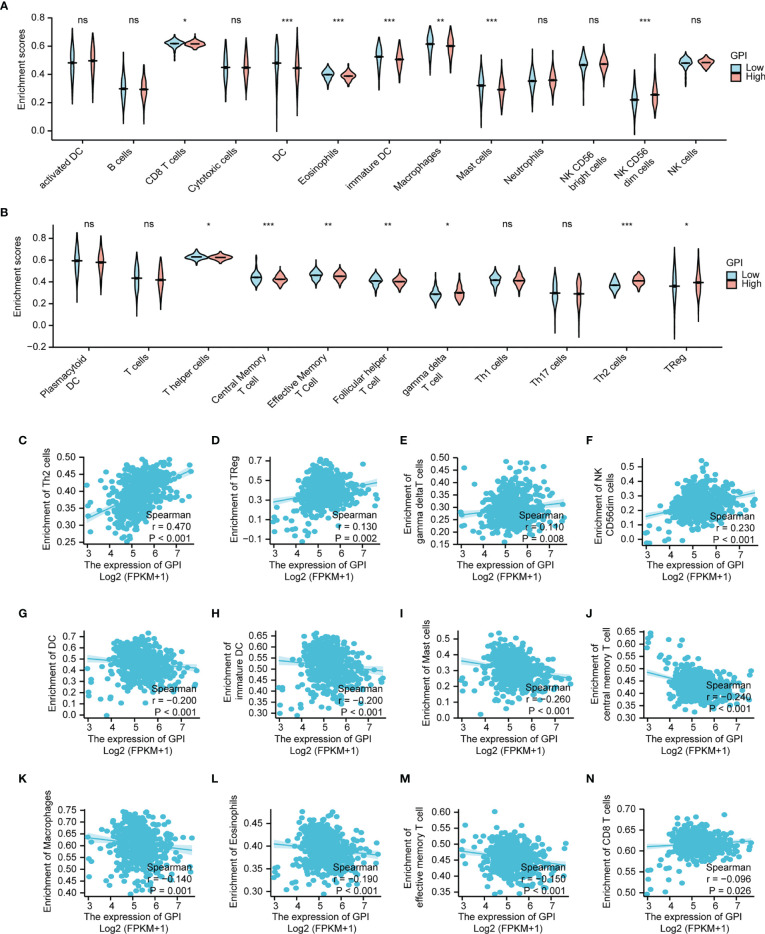
Correlation analysis of glucose-6-phosphate isomerase (GPI) expression and immune infiltration in lung adenocarcinoma (LUAD). **(A, B)** Differential distribution of immune cells in patients with high GPI expression and low GPI expression. **(C–N)** Correlation between the expression level of GPI and immune infiltration in lung adenocarcinoma (LUAD): **(C)** Th2 cells, **(D)** regulatory T cells, **(E)** gamma delta T cells, **(F)** NK CD56bright cells, **(G)** dendritic cells (DC), **(H)** immature DC, **(I)** Mast cells, **(J)** central memory T cells, **(K)** Macrophages, **(L)** Eosinophils, **(M)** effective memory T cell, **(N)** CD8+ T cells. *p < 0.05, **p < 0.01, ***p < 0.001, ns, no significance.

Research has shown that the levels of GPI are elevated in autoimmune diseases (26). In this study, we found that GPI is abnormally highly expressed in LUAD, so we speculate that GPI may be involved in regulating the tumor immune response. To explore the correlation between the expression level of GPI and tumor immune response, we used the TIMER database to investigate immune infiltration in LUAD with different GPI expression levels. The results showed that the infiltration levels of CD8^+^ T cells, dendritic cells (DCs), eosinophils, interdigitating cells, macrophages, mast cells, NK CD56 dim cells, central memory T cells, effective memory T cells, and follicular helper T cells in LUAD patients with high GPI expression were significantly lower than those in patients with low GPI expression. In contrast, the infiltration of Th2 cells, regulatory T cells (Tregs), and gamma delta T cells in LUAD patients with high GPI expression was significantly higher than that in patients with low GPI expression. In addition, there was no significant difference in the infiltration levels of Th1 cells, Th17 cells, B cells, natural killer (NK) cells, and neutrophils between patients with high and low GPI expression (**Figures 11A, B** **)**.

We further analyzed the correlation between the expression level of GPI and immune infiltration in LUAD, and the results showed that the expression level of GPI was positively correlated with the infiltrating levels of Th2 cells (*r* = 0.47, *p* < 0.001) (**Figure 11C**), Tregs (*r* = 0.13, *p* < 0.002) (**Figure 11D**), gamma delta T cells (r = 0.110, p =0.008) (**Figure 11E**) and NK CD56bright cells (r = 0.230, p <0.001) (**Figure 11F**). In contrast, the expression level of GPI was negatively correlated with the infiltrating levels of DC (r = -0.200, p < 0.001) (**Figure 11G**), immature DC (r = -0.200, p < 0.001) (**Figure 11H**), Mast cells (r = -0.260, p < 0.001) (**Figure 11I**), central memory T cells (r = -0.240, p < 0.001) (**Figure 11J**), (K) Macrophages (r = -0.140, p = 0.001) (**Figure 11K**), Eosinophils (r = -0.190, p < 0.001) (**Figure 11L**), effective memory T cell (r = -0.150, p < 0.001) (**Figure 11M**), CD8+ T cells (r = -0.0960, p = 0.026) (**Figure 11N**). A Th1/Th2 balance drift exists in many tumors, such as lung cancer, cervical cancer, breast cancer, gastric cancer, and other cancers (27). Th2 cells are often dominant, which may be related to tumor immune escape (28). Tregs also play an important role in tumor immune escape (29). Therefore, these data suggest that GPI may inhibit the tumor immune response of LUAD by positively regulating Th2 and Treg infiltration into tumors and negatively regulating CD8^+^ T-cell, central memory T-cell, DC, macrophage, mast cell, and eosinophil infiltration into tumors.

**Figure 12:**
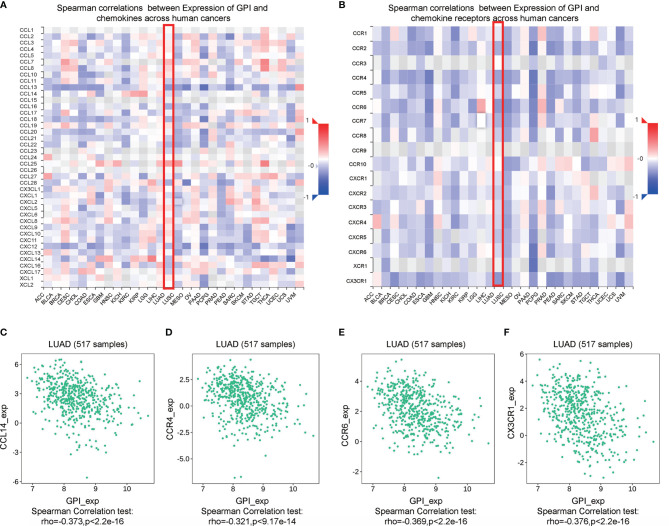
Correlation analysis between glucose-6-phosphate isomerase (GPI) expression and chemokines and/or chemokine receptors. **(A)** Heatmap analysis of the correlation between GPI and chemokines in tumors. **(B)** Heatmap analysis of the correlation between GPI and chemokine receptors in tumors. **(C–F)** GPI expression in lung adenocarcinoma (LUAD) is negatively correlated with CCL14, CCR4, CCR6, and CX3CR1.

Chemokines and chemokine receptors are essential for the infiltration of immune cells into tumors (30). Therefore, we used TISIDB to analyze the correlation between the expression level of GPI and immune cell chemokines and chemokine receptors in LUAD. The heatmap results showed that several chemokines and chemokine receptors were significantly correlated with the expression of GPI in LUAD (**Figures 12A, B** **)**. These results demonstrate that the GPI gene may play an important role in tumor immunity. To further clarify the relationship between GPI expression and immune cell migration, we comprehensively analyzed the correlation between GPI expression and chemokines/chemokine receptors. The results showed that GPI expression was negatively correlated with CCL14 (*r* = −0.373, *p* < 2.2e−16), CCR4 (*r* = −0.321, *p* < 9.17e−14), CCR6 (*r* = −0.369, *p* < 2.2e−16), and CX3CR1 (*r* = −0.376, *p* < 2.2e−16) (**Figures 12C–F**), whereas GPI was not highly correlated with other chemokines/chemokine receptors (−0.3 < *r* < 0.3). These data indicated that GPI is negatively correlated with the expressions of chemokines/chemokine receptors in LUAD.

**Figure 13:**
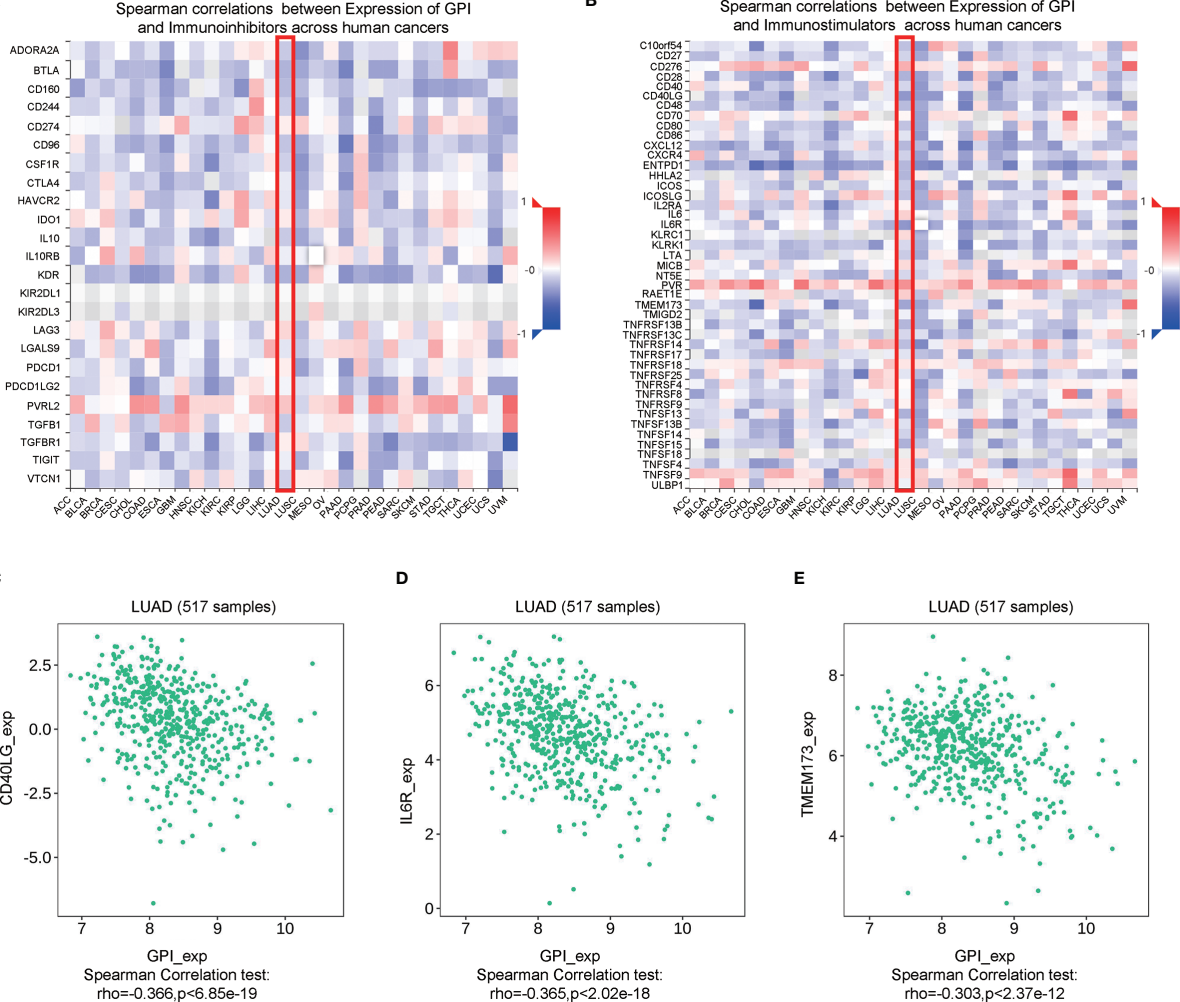
Correlation analysis between glucose-6-phosphate isomerase (GPI) expression and immunoinhibitors and immunostimulators. **(A)** Correlation between GPI and immunoinhibitors in tumors by heatmap analysis. **(B)** Correlation between GPI and immunostimulators in tumors by heatmap analysis. **(C–E)** GPI expression is negatively correlated with the immunostimulators CD40L, IL6R, and TMEM173 in lung adenocarcinoma (LUAD).

Immune checkpoint inhibitors (ICIs) are a significantly novel strategy for tumor immunotherapy that has gradually improved the prognosis of patients with many types of cancers (31). Subsequently, we analyzed the correlation between GPI and the expressions of immunoinhibitors and immunostimulators in different types of human cancers using the TISIDB database (**Figures 13A, B** **)**. Interestingly, there was no significant correlation between GPI and immunoinhibitor expression. However, GPI was negatively correlated with the expression of several immunostimulators, such as CD40L (*r* = −0.366, *p* < 6.85e−19), IL6R (*r* = −0.365, *p* < 2.02e−18), and TMEM173 (*r* = −0.303, *p* < 2.37e−12), in LUAD (**Figures 13C–E**). Therefore, these results suggest that GPI may play a role in regulating tumor immunity.

## Discussion

GPI encodes a member of the protein family of glucose phosphate isomerases. The encoded protein has been identified as a part-time protein based on its ability to perform mechanically different functions (10). In recent years, the role of GPI in tumors has gradually attracted attention. Studies have found that GPI is related to metastasis and poor prognosis in colorectal cancer, renal cell carcinoma, breast cancer, and endometrial cancer (13, 14, 32). At present, there are few studies on GPI in tumors. Considering the limited research on the GPI gene in cancer, we decided to conduct a fully integrated bioinformatics analysis to identify its biological function and potential regulatory pathway in LUAD. In this study, we first tried to determine the expression and prognostic value of the GPI gene in cancer and found that GPI expression was upregulated in LUAD. A high GPI expression is correlated with poor OS in LUAD. At the same time, a high GPI expression is related to poor clinicopathological characteristics. In addition, GPI has certain reference value for the diagnosis and prognosis of LUAD. These findings strongly suggest that GPI may be used as a biomarker for oncogenes and prognosis. To explain the underlying molecular mechanism of GPI affecting the prognosis of LUAD, we defined 56 genes from the GPI gene network as our hub genes, which can predict the survival rate and pathological stage of patients with LUAD. We also analyzed the expression and prognostic value of the GPI gene in lung squamous cell carcinoma (LUSC) and found that GPI expression was upregulated in LUSC, but not significantly associated with prognosis (data not shown).

Tumors are a type of disease characterized by uncontrolled cell growth and proliferation. Tumor cells are different from normal cells in terms of proliferation, differentiation, and apoptosis (33). Among these cells, cell cycle disorder is one of the most important features (34). In this study, to analyze the molecular mechanism of GPI-mediated LUAD progression and poor prognosis, we performed GO functional enrichment and KEGG pathway analysis and found that the cell cycle gene set was most significantly enriched in the GPI high-expression group. This conclusion was also verified by GSEA. To identify the underlying molecular mechanisms of GPI affecting the prognosis of LUAD, we defined 56 overlapping genes as our hub genes derived from crossing the 600 genes with the highest GPI correlation with the 148 survival-related upregulated genes in LUAD. GO functional enrichment and KEGG pathway analysis were used to analyze the biological processes and signaling pathways of the hub genes. Interestingly, cell cycle BP were the most enriched among the hub genes. The expression levels of cyclin genes in cancer cells are often different from those in normal cells (34). In this study, we found that GPI was positively correlated with the expressions of cell cycle regulation genes, such as *CCNA2*, *CCNB1*, *CCNB1*, and *CCNE1*. Our cell cycle experiments found that silencing GPI prevented LUAD cells from transitioning from the G2 phase to the M phase. Our data suggest that GPI may regulate the progression of LUAD by regulating the cell cycle transition from the G2 to the M phase.

The development of a cancer is related to its surrounding environment and tumor microenvironment (TME). The TME is composed of immune cells, the extracellular matrix, mesenchymal cells, and inflammatory mediators, which have an impact on tumor growth, metastasis, and clinical survival results (35). Although an effective immune response can have an antitumor effect, cancer cells have evolved a variety of mechanisms, including antigen presentation dysfunction and the recruitment of immunosuppressive cells, which promote tumors to evade the attack of immune cells. Previous studies have reported that immune infiltration can affect the prognosis of patients, and tumor-infiltrating lymphocyte grade is an independent predictor of sentinel lymph node status in patients with tumors (36). This study explored the correlation between the expression of GPI and the level of immune infiltration of LUAD. Our results showed that a high expression of GPI is positively correlated with the infiltration level of Th2 cells and Tregs; however, it is negatively correlated with the infiltration level of CD8^+^ T cells, DCs, macrophages, and eosinophils. These findings indicate that GPI may play an essential regulatory role in the tumor immune microenvironment and the development of LUAD.

Chemokines and their receptors play an important role in the directional migration of immune cells (37–40). In this study, the TISIDB database was used to analyze the correlation between the expression level of GPI and the expressions of immune cell chemokines and chemokine receptors in LUAD. The results showed that the expression level of GPI was negatively correlated with the expressions of CCL14, CCR4, CCR6, and CX3CR1, suggesting that a high expression of GPI may inhibit the migration of immune cells into the TME. Studies show that the active form of CCL14 binds to CCR1, CCR3, and CCR5 to promote the chemotaxis of monocytes, eosinophils, and T lymphocytes (41, 42). The inflammatory receptor CCR4 promotes the respiratory burst and phagocytosis of macrophages (43) and controls the differentiation of M1/M2 macrophages (44). CCR6 controls integrin-mediated adhesion in B cells (45, 46). CX3CR1 controls the survival of white blood cells and the activation of NK cells (47), which may explain how GPI regulates immune infiltration in LUAD.

In summary, our study found that the expression of GPI is significantly upregulated and is closely related to the poor prognosis of LUAD patients. GPI has certain reference value for the diagnosis and prognosis of LUAD. GPI may affect the progression of LUAD by regulating the cell cycle and immune infiltration. It may serve as a novel prognostic biomarker for LUAD patients.

However, even though we conducted a systematic analysis on GPI and used different databases for cross-validation, there were some limitations to this study. Firstly, the comparability and reproducibility of the microarray data generated in different laboratories remain controversial, which would lead to systemic bias. Secondly, *in vivo*/*in vitro* experiments are required to demonstrate the effect of GPI on the tumor cell cycle in order to improve the reliability of our results. Thirdly, even though we concluded that GPI expression is strongly related to immune infiltration and the prognosis of LUAD, we lack direct evidence that GPI influences prognosis by participating in immune infiltration. These issues are worth further exploration in the future.

## Data Availability Statement

The original contributions presented in the study are included in the article/**Supplementary Material**. Further inquiries can be directed to the corresponding authors.

## Ethics Statement

The studies involving human participants were reviewed and approved by the Institutional Research Ethics Committee of Taihe Hospital. The patients/participants provided written informed consent to participate in this study.

## Author Contributions

ZL and YY conceived and designed the work. JH and XD acquired, analyzed, and interpreted the data. RS, MLu, and MLi drafted and revised the article. BG, ZP, and YL helped with the cell culture and transfection. TZ and CT performed the cell cycle analysis and Western blot experiments. All authors contributed to the article and approved the submitted version.

## Funding

This study was supported by the National Nature Science Foundation of China (nos. 81802997 and 81602391), the Foundation for Free Exploration of Hubei University of Medicine (no. FDFR201802), the Natural Science Foundation of Hubei Province of China (nos. 2019CFA034, 2017CFB167, 2018CFB405, and 2017CFB456), the Natural Science Foundation of Hubei Provincial Department of Education (nos. Q20202106, D20172102, and Q20162109), and Hubei Provincial Health Commission (no. WJ2021M048).

## Conflict of Interest

The authors declare that the research was conducted in the absence of any commercial or financial relationships that could be construed as a potential conflict of interest.

## Publisher’s Note

All claims expressed in this article are solely those of the authors and do not necessarily represent those of their affiliated organizations, or those of the publisher, the editors and the reviewers. Any product that may be evaluated in this article, or claim that may be made by its manufacturer, is not guaranteed or endorsed by the publisher.
