# Supplementary material for: GPI Is a Prognostic Biomarker and Correlates With Immune Infiltrates in Lung Adenocarcinoma
Source: Front Oncol. 2021 Nov 29;11:752642. doi: 10.3389/fonc.2021.752642 (PMC8666546; doi:10.3389/fonc.2021.752642)
Supplement: Supplementary file 1 [file Table_1.docx]

**Supplementary Table 1. Methylation analysis of GPI in LUAD using DiseaseMeth version 2.0.**

| diseaseName | genomic region | transcript | gene | p-value | meanMethylDisea-meanMethylNormal |
| --- | --- | --- | --- | --- | --- |
| Lung adenocarcinoma | chr19:34854031-34856531 | NM_000175 | GPI | 0.053 | -0.023 |
| Lung adenocarcinoma | chr19:34853644-34856144 | NM_001184722 | GPI | 0.566 | 0.002 |
| Lung adenocarcinoma | chr19:34853644-34856144 | NM_001289789 | GPI | 0.566 | 0.002 |
| Lung adenocarcinoma | chr19:34854031-34856531 | NM_001289790 | GPI | 0.053 | -0.023 |
